# Transcription profiles of hydrogenases related genes in the cyanobacterium *Lyngbya majuscula *CCAP 1446/4

**DOI:** 10.1186/1471-2180-9-67

**Published:** 2009-04-07

**Authors:** Daniela Ferreira, Filipe Pinto, Pedro Moradas-Ferreira, Marta V Mendes, Paula Tamagnini

**Affiliations:** 1 IBMC – Instituto de Biologia Molecular e Celular, Universidade do Porto, Rua do Campo Alegre 823, 4150-180 Porto, Portugal; 2 Faculdade de Ciências, Universidade do Porto, Departamento de Botânica, Edifício FC4, Rua do Campo Alegre, s/n°, 4169-007 Porto, Portugal; 3 Instituto de Ciências Biomédicas Abel Salazar (ICBAS), Universidade do Porto, Largo Abel Salazar 2, 4099-003 Porto, Portugal

## Abstract

**Background:**

*Lyngbya majuscula *CCAP 1446/4 is a N_2_-fixing filamentous nonheterocystous strain that contains two NiFe-hydrogenases: an uptake (encoded by *hupSL*) and a bidirectional enzyme (encoded by *hoxEFUYH*). The biosynthesis/maturation of NiFe-hydrogenases is a complex process requiring several accessory proteins for e.g. for the incorporation of metals and ligands in the active center (large subunit), and the insertion of the FeS clusters (small subunit). The last step in the maturation of the large subunit is the cleavage of a C-terminal peptide from its precursor by a specific endopeptidase. Subsequently, the mature large and small subunits can assemble forming a functional enzyme.

**Results:**

In this work we demonstrated that, in *L. majuscula*, the structural genes encoding the bidirectional hydrogenase are cotranscribed, and that *hoxW *(the gene encoding its putative specific endopeptidase) is in the same chromosomal region but transcribed from a different promoter. The gene encoding the putative specific uptake hydrogenase endopeptidase, *hupW*, can be cotranscribed with the structural genes but it has its own promoter. *hoxH*, *hupL*, *hoxW *and *hupW *transcription was followed in *L. majuscula *cells grown under N_2_-fixing and non-N_2_-fixing conditions over a 12 h light/12 h dark cycle. The transcription of *hoxH*, *hoxW *and *hupW *did not vary remarkably in the conditions tested, while the *hupL *transcript levels are significantly higher under N_2_-fixing conditions with a peak occurring in the transition between the light and the dark phase. Furthermore, the putative endopeptidases transcript levels, in particular *hoxW*, are lower than those of the respective hydrogenase structural genes.

**Conclusion:**

The data presented here indicate that in *L. majuscula *the genes encoding the putative hydrogenases specific endopeptidases, *hoxW *and *hupW*, are transcribed from their own promoters. Their transcript levels do not vary notably in the conditions tested, suggesting that HoxW and HupW are probably constantly present and available in the cells. These results, together with the fact that the putative endopeptidases transcript levels, in particular for *hoxW*, are lower than those of the structural genes, imply that the activity of the hydrogenases is mainly correlated to the transcription levels of the structural genes. The analysis of the promoter regions indicates that *hupL *and *hupW *might be under the control of different transcription factor(s), while both *hoxH *and *xisH *(*hoxW*) promoters could be under the control of LexA.

## Background

Cyanobacteria are phototrophic prokaryotes that may contain up to two NiFe-hydrogenases, notably an uptake (encoded by *hupSL*) and a bidirectional enzyme (encoded by *hoxEFUYH*). *Lyngbya majuscula *CCAP 1446/4 is a N_2_-fixing filamentous nonheterocystous strain in which both hydrogenases are present [1-4]. The biosynthesis/maturation of NiFe-hydrogenases is a complex process, mediated by several accessory proteins, which assure the right assembly of metals and its ligands in the active center and in the electron transport clusters of the large and the small subunit, respectively. The last step in the maturation of the large subunit is the cleavage of a C-terminal peptide from its precursor. After this cleavage, the mature large subunit assembles with the mature small subunit and eventually the hydrogenase holoenzyme becomes active [5]. The genes encoding the hydrogenases accessory proteins were first characterized for *Escherichia coli*, and while most of these proteins affect the hydrogenases pleiotropically (Hyp proteins), the cleavage of the C-terminal peptide is processed by a specific endopeptidase [5,6]. Several genes presumably involved in the biosynthesis/maturation of cyanobacterial hydrogenases have been identified and characterized, in particular since cyanobacterial genome sequences became available [3,7-15]. In cyanobacteria, the *hyp *genes are frequently clustered and located in the vicinity of the structural genes of one of the hydrogenases, with a well known exception – the unicellular *Synechocystis *sp. strain PCC 6803 – in which *hypABCDEF *are scattered throughout the genome [for a review see [15]]. Recently, it was unequivocally demonstrated that *hypA1, B1*, *C*, *D*, *E *and *F *are required for an active bidirectional hydrogenase in *Synechocystis *sp. PCC 6803 [11]. The presence of a single copy of most of the *hyp *genes in cyanobacteria, regardless of possessing only the uptake hydrogenase (e.g. *Nostoc punctiforme*), the bidirectional hydrogenase (e.g. *Synechocystis *sp. PCC 6803) or both enzymes (e.g. *Nostoc *sp. PCC 7120) suggests that they might play a role in the maturation of both hydrogenases. The genes encoding the putative C-terminal hydrogenases-specific endopeptidases have been identified in several cyanobacteria, and were named *hupW *(gene putatively encoding the enzyme processing the uptake hydrogenase) and *hoxW *(gene putatively encoding the enzyme processing the bidirectional hydrogenase) [3,11,16-19]. However, so far only Hoffmann et al. [11] reported the construction of a cyanobacterial endopeptidase deficient mutant, demonstrating that *hoxW *is required for the bidirectional hydrogenase activity in *Synechocystis *sp. PCC 6803. Since this cyanobacterium possesses only the bidirectional hydrogenase, studies on strains containing only the uptake or both enzymes are required to prove the actual involvement and specificity of the endopeptidases, HoxW and HupW, as well as biochemical evidence on the role of the two proteins as endopeptidases. Yet, the pattern found in other organisms, and the fact that *hupW *and *hoxW *are present only in strains containing both the uptake and the bidirectional hydrogenase, suggests that each gene encodes the protease specific for one of the hydrogenases [15,19].

The position of *hupW *and *hoxW *in the cyanobacterial chromosome is variable; however, in some cases they are located in the proximity of the corresponding hydrogenase structural genes [15]. In *Gloeothece *sp. ATCC 27152, *hupW *is immediately downstream and is cotranscribed with *hupSL *[17]. Similarly, in *Synechococcus *sp. PCC 6301 and in *Synechococcus *sp. PCC 7942 *hoxW *is part of a transcriptional unit containing *hoxUYH*, but in the last strain it is mainly expressed by its own promoter [16,18]. Not much is known about the transcription patterns of the genes encoding the putative hydrogenases specific endopeptidases, nevertheless it was shown that *hupW *was transcribed under N_2_- and non-N_2_-fixing conditions in the heterocystous cyanobacteria *N. punctiforme *and *Nostoc *sp. PCC 7120, strains harboring only the uptake or both hydrogenases, respectively [19]. These authors hypothesize that the transcription of *hupW *under conditions in which *hupSL *are not transcribed could indicate a constitutive expression of *hupW*. Until date there is no information on the transcription patterns of the structural versus endopeptidases genes on filamentous non-heterocystous cyanobacteria. Therefore, in this work besides pursuing the characterization of the *hox *genes in *L. majuscula *CCAP 1446/4, we evaluated the concomitant transcription of the hydrogenases structural genes – *hupL *and *hoxH *– with the genes encoding their putative respective putative C-terminal endopeptidases – *hupW *and *hoxW*.

## Results

### Physical organization of the *hox *genes

**Figure 1:**
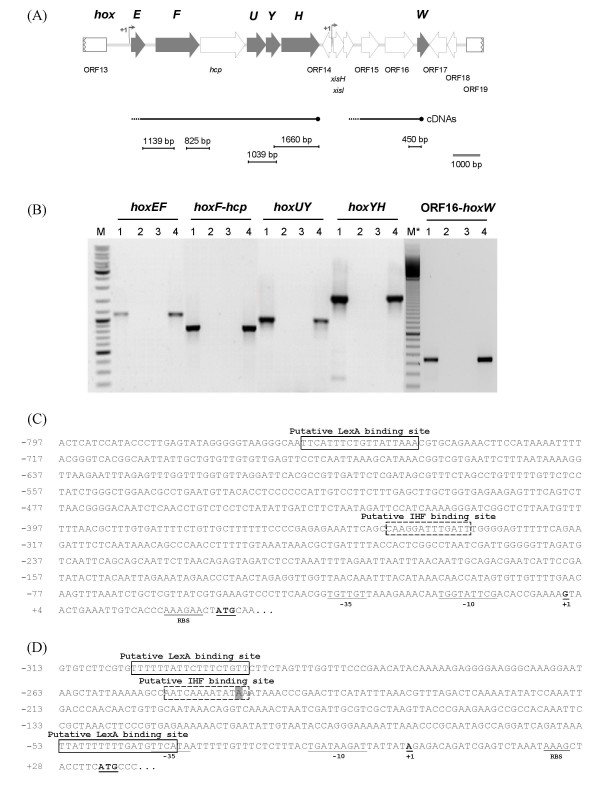
***hox *genes physical map, *hoxE *and *xisH *promoters, and analysis of cotranscription in *Lyngbya majuscula *CCAP 1446/4**. (A) Physical map of the *L. majuscula *genome region containing the *hox *genes, (B) analysis of the *hox *genes cotranscription by RT-PCR, and (C, D) nucleotide sequences of the promoter regions upstream of *hoxE *and *xisH*. A schematic representation of the cDNAs and the products generated in the RT-PCRs are depicted below the physical map. Lanes 1: RT-PCR; Lanes 2: Negative control without reverse transcriptase; Lanes 3: Negative control (no template); Lanes 4: PCR positive control (genomic DNA); M: GeneRuler DNA Ladder mix (Fermentas). M*: 100 Base-Pair Ladder (GE Healthcare). Within the *hoxE *and *xisH *promoter regions the following regions are indicated: putative LexA binding sites, putative IHF binding sites (boxed with the mismatching nucleotide shaded), the -10 and -35 boxes and the ribosome binding site – RBS (underlined), the transcription start point (+1, bold and underlined), and the start codons of *hoxE *and *xisH *(bold and underlined).

In *Lyngbya majuscula *CCAP 1446/4 the five structural genes encoding the bidirectional hydrogenase, *hoxEFUYH*, are clustered and orientated in the same direction (Fig. 1A), with *hcp*, encoding a putative hybrid cluster protein, between *hoxF *and *hoxU*. The 14764 bp region sequenced includes several other ORFs downstream of the *hoxH*, the first one in the opposite direction compared to the *hox *cluster (Fig. 1A). Among these ORFs, and ca. 3.5 kb downstream from *hoxEFUYH*, a gene encoding the putative bidirectional hydrogenase-specific endopeptidase (*hoxW*) can be discerned. This sequence is available from GenBank under accession number AY536043. The proteins predicted to be encoded by the identified ORFs, as well as the respective putative functions and/or characteristics, are listed in Table 1, with the exception of ORF15 and ORF16 for which no homologues were found in the database, even when compared with the available cyanobacterial genomes.

**Table 1:** Predicted function and/or characteristics of the putative proteins encoded by the ORFs present in the *hox *chromosome region of *Lyngbya majucula *CCAP 1446/4

ORF	Putative function/characteristics of the encoded protein
ORF13 (partial)	POR_N, pfam01855: Pyruvate flavodoxin/ferredoxin oxidoreductase, thiamine diP-dinding domain; belongs to NifJ (nitrogen fixation) family
*hoxE*	PRK07571: Bidirectional hydrogenase complex protein HoxE
*hoxF*	PRK11278: NADH dehydrogenase I subunit F
*Hcp*	cd01914: Hybrid cluster protein (prismane protein); hydroxylamine reductase activity and possible role the nitrogen metabolism; specific function unknown
*hoxU*	PRK07569: Bidirectional hydrogenase complex protein HoxU
*hoxY*	COG3260: NiFe-hydrogenase small subunit
*hoxH*	COG3261: NiFe-hydrogenase large subunit
ORF14	Hypothetical protein; 3 predicted transmembrane helixes
*xisH*	pfam08814: XisH, required for excision of a DNA element within *fdxN*
*xisI*	pfam08869: XisI, required for excision of a DNA element within *fdxN*
ORF15	Hypothetical protein; no putative conserved domains detected, nor relevant homologies found in cyanobacteria
ORF16	Hypothetical protein; no putative conserved domains detected, nor relevant homologies found in cyanobacteria
*hoxW*	COG0680: NiFe-hydrogenase maturation factor cl00477: HycI, hydrogenase maturation protease
ORF17	DUF820, pfam05685: hypothetical protein; conserved in cyanobacteria COG4636, Uma2 family: Restriction endonuclease fold
ORF18	COG4067: hypothetical protein; conserved in Archaea [Posttranslational modification, protein turnover, chaperones] DUF785, pfam05618: hypothetical protein
ORF19 (partial)	DUF1400, pfam07176: Alpha/beta hydrolase of unknown function

### Cotranscription of *hoxEFUYH *and *hoxW*, and *hupSL *and *hupW*

**Figure 2:**
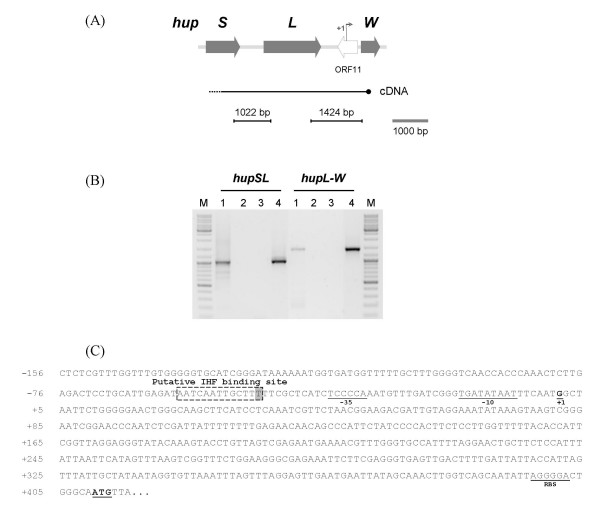
***hup *genes physical map, *hupW *promoter, and analysis of cotranscription in *Lyngbya majuscula *CCAP 1446/4**. (A) Physical map of *L. majuscula hup *genes (adapted from [3], accession number GenBank:AF368526), (B) analysis of the *hup *genes cotranscription by RT-PCR, and (C) nucleotide sequence of the promoter region upstream of *hupW*. A schematic representation of the cDNA and the products generated in the RT-PCRs is depicted below the physical map. Lanes 1: RT-PCR; Lanes 2: Negative control without reverse transcriptase; Lanes 3: Negative control (no template); Lanes 4: PCR positive control (genomic DNA); M: GeneRuler DNA Ladder mix (Fermentas). Within the *hupW *promoter region the following regions are indicated: a putative IHF binding site (boxed with the mismatching nucleotide shaded), the -10 and -35 boxes and the ribosome binding site – RBS (underlined), the transcription start point (+1, bold and underlined), and the start codon of *hupW *(bold and underlined).

To assess the cotranscription of *hox *genes and to clarify if the genes encoding the hydrogenases-specific endopeptidases (*hoxW *and *hupW*) are cotranscribed with the respective structural genes, RT-PCR experiments were performed with RNA collected from *Lyngbya majuscula *cells grown in conditions in which the transcript levels were demonstrated to be high (for details see Material and Methods, [1,2]). The cDNAs were synthesized using a *hoxH*-, a *hoxW*- or a *hupW*-specific antisense primer, and amplifications were performed with primer pairs that covered regions between *hoxEF*, *hoxF-hcp*, *hoxUY*, *hoxYH*, ORF16-*hoxW*, *hupSL *and *hupL-W*. In all cases, PCR products were obtained (Fig. 1A, B, 2A and 2B). These data indicate that all the structural genes encoding the bidirectional hydrogenase, and the gene putatively encoding the hybrid cluster protein (*hcp*), can be transcribed as a single operon in *L. majuscula*. The results also show that *hoxW *is cotranscribed with ORF16 (Fig. 1B), ORF15, *xisI *and *xisH *(data not shown). The ORF14 is in the opposite direction in relation to the *hox *genes, and no PCR product was detected using the cDNA generated with *hoxW*-specific primer and ORF14 specific primers. In order to assess the transcription of ORF14, RT-PCR was performed using cDNA synthesized with random primers, the only PCR product obtained was generated using a ORF14 internal primer pair suggesting that ORF14 is indeed transcribed as a monocistronic unit (data not shown). Concerning the uptake hydrogenase it has been previously demonstrated that the structural genes *hupSL *were cotranscribed [2], however until now the transcription of the gene encoding the putative specific endopeptidase -*hupW *– was not accessed. In this work, we demonstrated that *hupW *can be transcribed together with *hupSL*, although a promoter region upstream *hupW *was also identified (see Fig. 2C and text below).

### Transcriptional start site mapping and promoter analysis

The transcription start point (tsp) of the bidirectional hydrogenase structural genes was identified 27 bp upstream from the *hoxE *start codon, and analysis of the upstream region revealed at least one putative binding site for LexA, and one for the integration host factor (IHF), in addition to the presence of an extended -10 box [20-22] and a -35 box. Moreover, a putative Shine-Dalgarno sequence (ribosome-binding site; RBS) could be discerned immediately upstream *hoxE *(Fig. 1C).

Using 5'RACE no tsp could be detected immediately upstream *hoxW*, ORF16, ORF15 or *xisI *but one tsp was identified 33 bp upstream the *xisH *start codon. Analysis of the *xisH *putative promoter region revealed the presence of putative LexA and IHF binding sites, an extended -10 box, -35 box, and a putative RBS (Fig. 1D).

*L. majuscula *uptake hydrogenase structural genes (*hupSL*) were previously characterized, and their promoter region analysed by Leitão *et al*. [2]. Subsequently, the putative uptake hydrogenase-specific endopeptidase gene, *hupW*, was also identified 1102 bp downstream of *hupL *[3]. Within this work we demonstrated that *hupW*, even though possibly cotranscribed with *hupSL*, has his own promoter region (Fig. 2C), with a tsp located 409 bp upstream from the start codon. The analysis of this region revealed the presence of a putative IHF binding motif, an extended -10 box, as well as a -35 box, both regions separated exactly by 17 bp, a consensus length that has been established for this spacer [21]. Moreover, a putative RBS could also be identified in the 5'UTR of *hupW *(Fig. 2C).

### Transcription profiles of hydrogenases structural genes and respective endopeptidases genes

**Figure 3:**
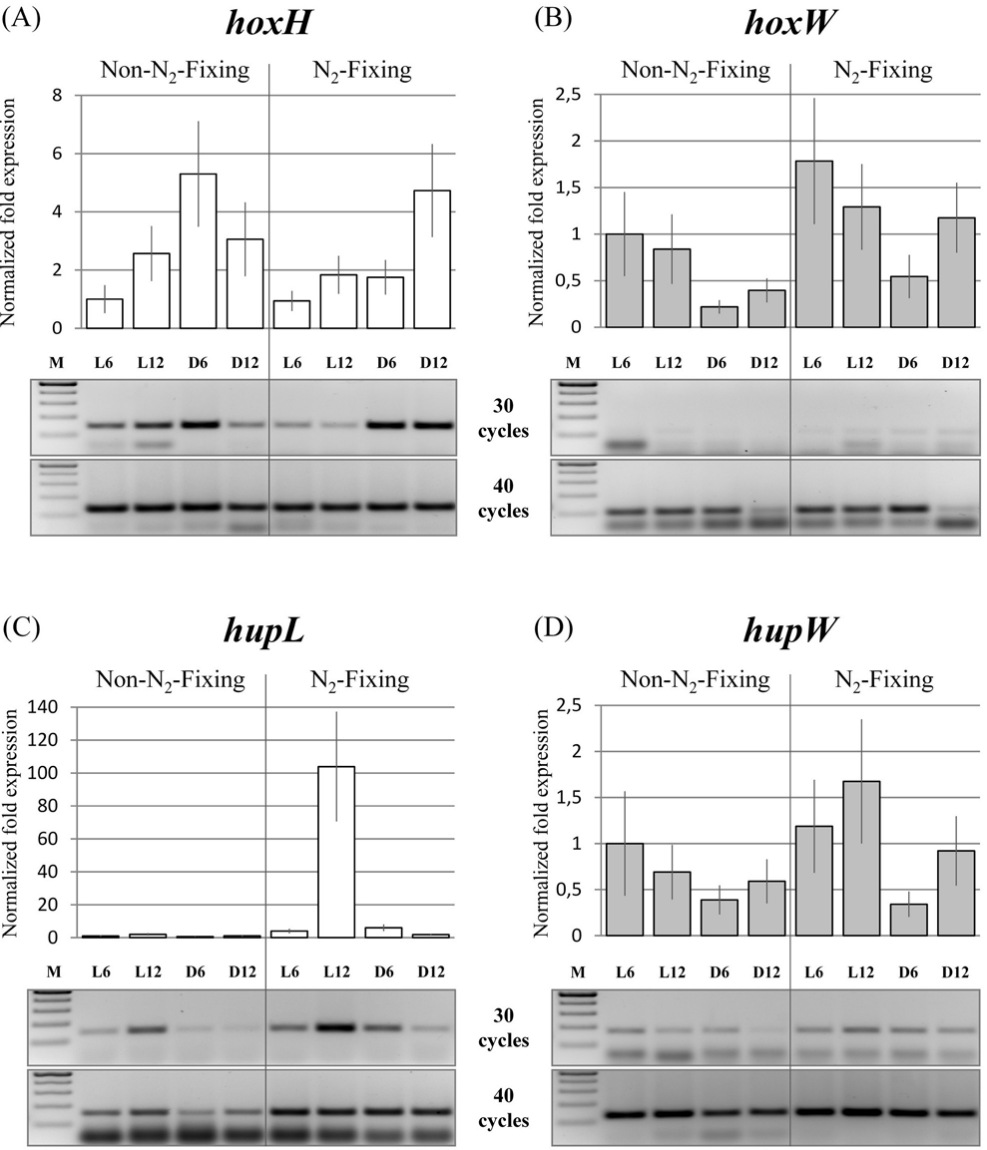
**Transcription profiles of the hydrogenases structural genes versus the putative specific endopeptidases genes in *Lyngbya majuscula *CCAP 1446/4**. Transcription profiles of *hoxH *(A), *hoxW *(B), *hupL *(C), and *hupW *(D) genes in *L. majuscula*, evaluated by Real-time RT-PCR (graphs) and RT-PCR (pictures below). The filaments were grown in N_2_-fixing or non-N_2_-fixing conditions during a 12 h light/12 h dark cycle, and the samples were collected at 6 h intervals during a complete 24 h cycle (L6 and L12 – light samples, D6 and D12 – dark samples). The cDNAs were produced with random primers, and used in PCR amplifications performed with specific primer pairs (see Methods). For the Real-time experiments, the Mean Normalized Expression (± standard errors) of the target genes was calculated relative to the transcription of the reference gene (16S rDNA) and the reaction internal normalization was performed using the sample L6 from non-N_2_-fixing conditions. In the RT-PCRs two sets of experiments were performed using 30 and 40 cycles, and the 16S rDNA detection is not shown.

The transcription of the structural genes encoding the large subunits of the bidirectional and the uptake hydrogenase, and their putative respective C-terminal specific endopeptidases – *hoxH*, *hupL*, *hoxW*, and *hupW *– was followed in *L. majuscula *cultures grown under N_2_-fixing and non-N_2_-fixing conditions over a 12 h light/12 h dark cycle, using Real-time RT-PCR and RT-PCR. The transcription of *hoxH *did not vary notably in the two conditions tested (N_2_-fixing and non-N_2_-fixing), yet an increase in the transcript levels can be observed during the dark periods (Fig. 3A). In contrast, significant higher levels of *hupL *transcript can be detected under N_2_-fixing conditions compared to non-N_2_-fixing conditions, with the maximum occurring in the transition between the light and the dark phase (Fig. 3C). This peak is particularly evident under N_2_-fixing conditions (Fig. 3C, right panel). During the 24 hours cycle and in the two conditions tested, the transcript levels of the genes encoding the putative specific endopeptidases – *hoxW *and *hupW *– do not vary significantly (Fig. 3B and 3D). Furthermore, it can be observed that the endopeptidases transcript levels are lower than those of the respective hydrogenase structural genes, in particular for *hoxW *(Fig. 3). The data from RT-PCR (higher number of cycles required for detection of the transcripts) are confirmed by the Ct values obtained in the Real-time experiments (data not shown).

## Discussion

### *hox *genes chromosome region and putative encoded proteins

In cyanobacteria, the structural genes encoding the bidirectional hydrogenase are organized in a dissimilar way [15]. The organization of the *hox *operon in *Lyngbya majuscula *CCAP 1446/4 resembles one of the two patterns previously reported with the *hoxEFUYH *genes grouped with a few ORFs interspersed [12,23,24], and contrasts with the arrangement into two different clusters, with *hoxEF *and *hoxUYH *separated by several kb, observed in strains like *Synechococcus *sp. PCC 6301 and *Nostoc *sp. PCC 7120 [25,26]. In *L. majuscula *a single gene encoding a hybrid cluster protein is present in the middle of the bidirectional hydrogenase structural genes. *hcp *homologues are present among *hox *genes in other filamentous nonheterocystous strains, notably in *L. aestuarii *CCY 9616 and *Arthrospira platensis *FACHB341, but not in unicellular and heterocystous strains where the *hcp *can be found in other regions of the chromosome.

Similarly, most of the other ORFs found in the vicinity of the *hox *genes in *L. majuscula*, with the exception of ORF15 and ORF16, have homologues in other cyanobacterial genomes, but they are not necessarily present in the *hox *region. Yet, in the closely related strain, *L. aestuarii *CCY 9616, homologues of ORF13, ORF14, ORF17, ORF18 and ORF19 can be found exactly in the same position and direction as in *L. majuscula *(L8106_07471, L8106_07436, L8106_07426, L8106_07421 and L8106_07416, respectively). Upstream of *hoxE*, the protein encoded by the partially sequenced ORF13 contains a pyruvate flavodoxin/ferredoxin oxidoreductase domain.

The gene immediately downstream of *hoxH*, ORF 14, encodes a protein containing three transmembrane α-helices predicted by TMHMM2.0 http://www.cbs.dtu.dk/services/TMHMM/. ORF14 also shows homology to cyanobacterial genes coding for putative membrane proteins. The following genes, named *xisH *and *xisI*, have homologues in several cyanobacterial strains, and although it has been demonstrated that they are required for the heterocyst-specific excision of the *fdxN *element (*fdxN *encodes a heterocyst-specific ferredoxin) in *Nostoc *sp. PCC 7120 [27], they have been found in several unicellular and nonheterocystous strains, as in the case of *L. majuscula*. In the nonheterocystous strains the function of the proteins encoded by *xisH *and *xisI *is still to be disclosed.

The three ORFs identified downstream of *hoxW*, have homologues in other cyanobacterial genomes, nevertheless the function of the encoded proteins is not known.

### Putative hydrogenase-specific endopeptidases genes and proteins

In *L. majuscula*, the genes encoding the putative hydrogenase-specific endopeptidases, *hoxW *and *hupW*, are in the vicinity of the respective hydrogenases structural genes as it is common for cyanobacteria [3,15-18]. The deduced 152 amino acid sequence of *L. majuscula *HoxW shows homology with the corresponding sequences of cyanobacteria with values varying between 32% and 82% of identity. In contrast, the deduced amino acid sequence of HupW from *L. majuscula *shows 59% to 80% of identity compared to the corresponding cyanobacterial sequences, being overall much less variable than HoxW. HoxW and HupW from *L. majuscula *exhibit only 23% identity between themselves, a range that is frequent for other cyanobacterial strains. This low homology might be related to the specificity of the endopeptidases towards the hydrogenases large subunits, a subject that needs further investigation.

### Promoter regions and transcription of the *hox *genes

In *L. majuscula*, *hoxEF-hcp-hoxUYH *are transcribed as an operon, as it could be predicted by the physical organization of the genes in a single cluster. In agreement with the different patterns of organization, the cyanobacterial *hox *genes can be transcribed as one or several units depending on the strain [15,16,18,28-30]. *L. majuscula hoxW*, is not cotranscribed with the bidirectional hydrogenase structural genes or ORF14 but it is transcribed together with the four ORFs immediately upstream (*xisH*, *xisI*, ORF15 and ORF16), and its transcription is most probably controlled by the *xisH *promoter. Once more, in cyanobacteria the transcription patterns are complex and strain specific, and *hoxW *can also be transcribed from its own promoter [15], together with *hoxUYH *[16] or from both promoters [18].

The analysis of *L. majuscula hoxE *and *xisH *promoter regions, revealed putative binding sites for LexA, using the motif described by Domain *et al*. [31], and for the integration host fact IHF. It was previously demonstrated that LexA is a transcriptional regulator of the *hox *genes in *Synechocystis *sp. PCC 6083 and *Nostoc *sp. PCC 7120 [28-30], acting as an activator in *Synechocystis *sp. PCC 6803 [28].

Additionally, LexA was also suggested to be involved in the transcriptional regulation of *hyp *genes, encoding the proteins putatively involved in the biosynthesis/maturation of hydrogenases in *L. majuscula *[1]. Recently, besides LexA, an AbrB-like protein was shown to specifically interact with the *Synechocystis *sp. PCC 6803 *hox *promoter region activating the transcription [32]. However, putative recognition motifs for the AbrB-like protein are not yet described. IHF has been described to act together with other transcription factors providing an appropriate deformation of the DNA scaffold activating transcription [33,34]. Consequently, it is possible that the binding of IHF to the *hoxE *and *xisH *promoter regions will promote the bending of the DNA, favouring the contact between the transcription factors associated upstream (LexA) and the RNA polymerase complex.

### Promoter region and transcription of *hupW*

It has been previously described that, similar to other cyanobacteria, the *hupSL *genes are cotranscribed in *L. majuscula *[2,15]. However, the cotranscription of *hupSLW *has been demonstrated only for *Gloeothece *sp. ATCC 27152 [17], while in *Nostoc *sp. PCC 7120 and *N. punctiforme hupW *seems to be transcribed independently from *hupSL *[19]. In *L. majuscula*, the RT-PCR data shows that *hupL *might be cotranscribed with *hupW *but the identification of a transcription start point upstream of *hupW *suggests that this gene is also transcribed from its own promoter. This is not the first time that the existence of different transcripts for the structural hydrogenase genes and its putative specific C-terminal endopeptidase is reported, since it has previously been shown that *hoxW *can be part of a transcriptional unit containing *hoxUYH*, but it is mainly transcribed from its own promoter in *Synechococcus *sp. PCC 7942 [18].

In *L. majuscula*, a putative IHF binding site was found in the *hupW *promoter region, similar to what was reported for the *hupSL *promoter [2]. It was previously shown that the transcriptional factor NtcA, a protein that operates global nitrogen control in cyanobacteria [35], binds the *hupSL *genes promoter region of several cyanobacteria, including *L. majuscula *[2,15,36], but no NtcA consensus sequence signature could be recognized in the *L. majuscula hupW *promoter. It is important to retain that in *L. majuscula *it was demonstrated that NtcA binds also to the *hyp *cluster promoter region, indicating that in this organism NtcA is involved in the regulation of the structural genes encoding the uptake hydrogenase and in the regulation the genes encoding proteins pleiotropically involved in the biosynthesis/maturation of hydrogenase(s) but does not seem to control directly the transcription of the specific endopeptidase.

### Transcription profiles: structural versus hydrogenase specific endopeptidases genes

In order to compare the transcription profiles of *hoxW *and *hupW *with *hoxH *and *hupL*, Real Time RT-PCR and RT-PCR assays were performed with RNA extracted from cells grown in conditions previously tested and in which was possible to see fluctuations in the transcript levels of *hoxH *and *hupL *[1,2]. The *hoxH *transcript levels do not vary significantly in the conditions tested, but a minor increase can be observed in the dark phase of either N_2_- or non-N_2_-fixing conditions. These results are in agreement with the observations of Ferreira *et al*. [1] and can be explained by the decline of the intracellular O_2 _levels. Although the physiological function of the cyanobacterial bidirectional hydrogenases is still unclear, the influence of the intracellular O_2 _pressure would be expected. It has been proposed that this enzyme plays a role in dark fermentative processes [37], or it acts as an electron valve during photosynthesis [38]. Therefore, the role of this enzyme could be influenced by the redox state of the cell. Indeed, in the purple sulfur phototrophic bacterium *Thiocapsa roseopersicina*, a redox control of its "cyanobacterial-type" soluble bidirectional hydrogenase has been suggested [39]. Moreover, a positive influence of microaerobic/anaerobic conditions in the *hox *transcription and the enzyme activity has been demonstrated for several heterocystous cyanobacteria [30,40-45]. Nitrogen limited conditions have also been reported as increasing the bidirectional hydrogenase activity in *Gloeocapsa alpicola *CALU 743 and *Synechocystis *sp. PCC 6803, but only in the later strain an increase was observed at the transcriptional level [4,32,45,46]. With this work we confirmed that in *L. majuscula *the nitrogen source (N_2 _versus ammonia) does not affect the *hox *transcript levels as previously suggested by Ferreira *et al*. [1].

The amount of transcripts of *hoxW *is considerably lower than those of the respective hydrogenase's large subunit, and the levels do not vary much along the 24 hours cycle and with the conditions tested. In agreement, it was previously demonstrated that both *hoxH *and *hoxW *are transcribed under N_2_- and non-N_2_-fixing in the heterocystous cyanobacterium *Nostoc *sp. PCC 7120, a strain also harboring the two hydrogenases [19]. In both *L. majuscula *and *Nostoc *sp. PCC 7120 the bidirectional hydrogenase structural genes and *hoxW *are not cotranscribed, and since transcripts are present in all the conditions tested it is difficult to infer if they are or are not independently regulated. In contrast with the results obtained here for *L. majuscula*, in *Synechococcus *sp. PCC 7942 the *hoxW *transcript levels were higher compared to the *hoxH *[18].

It has been previously demonstrated that for *L. majuscula *cells grown under N_2_-fixing conditions and 12 h light/12 h dark regimen, the maximum transcript levels of *hupL *occurred in the transition between the light and the dark phase [1,2], and that a substantial decrease occurred under non-N_2_-fixing conditions although the transcription/expression was not completely abolished even in the presence of ammonium [1]. The results obtained in this work for the transcription of *hupL *confirm the pattern reported previously, whereas the *hupW *transcript levels did not vary significantly in the two conditions tested (although slightly higher in N_2_-fixing conditions). Similarly, for the heterocystous *Nostoc *sp. PCC 7120 and *Nostoc punctiforme*, it was demonstrated that *hupW *is transcribed under both N_2_- and non-N_2_-fixing conditions [19]. At the time, the authors postulated that the transcription of *hupW *in conditions in which *hupL *transcripts are not detected (non-N_2_-fixing conditions) could imply that *hupW *is constitutively expressed and independently transcribed from the uptake hydrogenase structural genes. In contrast, in the unicellular strain *Gloeothece *sp. ATCC 27152 *hupW *was shown to be cotranscribed with *hupSL *[17], however it was not accessed if *hupW *is transcribed under non-N_2_-fixing conditions. In this work, the experiments performed with *L. majuscula *revealed that although *hupW *can be cotranscribed with *hupSL *it has its own promoter, and the dissimilar transcription patterns, observed for these genes, indicate that the *hupSLW *transcript is rare. This is supported by previous studies, in which a Northern blot analysis using a *hupL*-specific probe, showed a transcript size that corresponds to *hupSL *and not to *hupSLW *[2].

## Conclusion

The number of transcriptional studies regarding the genes encoding the putative cyanobacterial hydrogenases-specific endopeptidases is still too limited to infer specific transcription pattern(s) for this group of organisms. The data presented here suggest that in *L. majuscula hoxW *and *hupW *are transcribed from their own promoters and that there are minor fluctuations in the transcript levels in the conditions tested, being HoxW and HupW probably constantly present and available in the cell. Since the putative endopeptidases genes transcript levels, in particular *hoxW*, are lower than those of the structural genes, one may assume that the activity of the hydrogenases is mainly correlated to the transcription levels of the structural genes. The analysis of the promoter regions indicates that *hupL *and *hupW *might be under the control of different transcription factor(s), while both *hoxH *and *xisH *(*hoxW*) promoters contain LexA-putative binding sites in *L. majuscula*. However, it is important to retain that the identification of the factors involved in the regulation of the genes related to cyanobacterial hydrogenases is still in its infancy and far from being elucidated.

## Methods

### Strains and culture conditions

The marine filamentous cyanobacterium *Lyngbya majuscula *CCAP 1446/4 (Culture Collection of Algae and Protozoa, Scotland, UK) was grown in either BG11_0 _or BG11_0 _supplemented with 5 mM NH_4_Cl and 10 mM HEPES (pH 7.5) [47] at 25°C, on a 12 h light (7 μmol photons/m^2^/s)/12 h dark regimen. For cloning purposes *Escherichia coli *strain DH5α (Stratagene, La Jolla, CA) was used. *E. coli *cells were grown at 37°C on selective LB (Luria-Bertani) medium.

### DNA and RNA extraction, PCRs, and DNA recovery

Cyanobacterial genomic DNA was extracted using the phenol-chloroform method described previously [48]. For RNA extraction *L. majuscula *filaments were collected at six hours intervals during a complete 24 h light/dark cycle, and frozen at -80°C. RNA was extracted using the TRIZOL^® ^Reagent (Invitrogen Corporation, Carlsbad, CA) according to the method described previously [3]. PCRs were carried out in the thermal cycler MyCycler™ (Bio-Rad Laboratories, Inc., Hercules, CA) using the conditions described previously [48]. The oligonucleotide primers used in this study are listed in Table 2. Agarose gel electrophoresis was performed by standard protocols using 1× TAE buffer [49], and the DNA fragments were isolated from gels using the GFX™ PCR DNA and Gel Band Purification Kit (GE Healthcare, Buckinghamshire, UK), according to the manufacturer's instructions.

**Table 2:** Oligonucleotide primers used in this study

Primer^*a*^	Sequence 5' → 3'	Primer^*a*^	Sequence 5' → 3'
**GWhox1R**	ATCAGCACCTCGTCCAGCAACATCCC	**LmhupW1R**	CGCAGTTCCGCAGTCAAAGATTCGCA
**GWhox2F**	CGCTAAGTTACCCGAAGAAGCCGCCAC	**LmhupW2R**	TCAAAAACTGCACCGGGTTCT
**GWhox3R**	CAACTTCTCGCTTAGGAAATATGTAGGG	**LmhupW3R**	GGTTCGGGTAATTGTTCAAGCTCT
**GWhox4R**	CTAAATGACAGAGGACGGGAATATGAACC	**LmHCPR**	GGTTGATAACAACTGATCTCAGACCAT
**GWhox5R**	ACCTTCTTCTACGGCAACGTCAATGTCG	**LmhoxHF^*d*^**	CGTGCGATCGCCCTTATCAGAAGA
**GWhox6F**	GAGACCTCTATCACGATACTGTCCGCAC	**LmhoxHR^*d*^**	AGTTAGAATTAATTGCGGAAAACCTC
**GWhox7R**	TAAGCCAATATCTTGACCCTGTTGTGC	**LmhoxUF1**	CGACATTGACGTTGCCGTAGAAGAAGG
**GWhox8R**	GATGGGGATCACTTTCAAGGACACTGCG	**LmhoxYF**	GATGTCACGCCGCAAATTCCGAAA
**GWhox9R**	GAAGGGACTTTCACGATAACGAGCAG	**LmhoxWorfF1**	TTATTCAAACTTTACGCAAGTCAAGGT
**GWhox10F**	AGGAAAGGTAGTAGTGAAACAACCTG	**LmhoxWR2**	CTGATGGAGTGCTAAACATTTCACAT
**GWhoxW1F**	CTCCATCAGTTGCTTCCTGAAGTTG	**LmxisHR1**	CAAATCTACAAACGTGGAACGTCC
**GWhoxW1R**	GATGTCCCCAGTTAAGTTCAGTTGTTTC	**LmxisHR2**	GCAAGATAAAGATAACGGTCTGGT
**GW5Lmhox2R**	TTTGCGGCGTGACATCACCTAACTCTA	**LmxisHR3**	CGTCTGTAATTGTCCAACCCTCTT
**GW3LmhupWR1**	TTTAGATTCCCACAACCAATAATCG	**LmxisHR4**	AATCTCGTTCAGCTGCAATGAGTT
**LahoxWF1**	TTCGGGGTGATGATGG	**RChoxE1R**	ACCAGGGAAACTAAACCGTC
**LahoxWR1**	GGAACTGATATTAACCAAGC	**RChoxE2R**	TGAGGGAAAACAAATGGTAAAAGG
**BD16SF1**	CACACTGGGACTGAGACAC	**RChoxE3R**	GGAAGTTTGAGGTTACGGGCGAC
**BD16SR1**	CTGCTGGCACGGAGTTAG	**RChoxE4R**	GGTAAGGCACGGTTATTCC
**BDhoxHF1**	GATGATGCGGGCGAAGTTG	**RThoxE1F**	TTGGGGCTTGTGGGATTGC
**BDhoxHR1**	AGCGAGTAGGTGACTAACGG	**RThoxE2F**	TGAAACGCAGCCAATATCGT
**BDhupLF1**	ACACAAGCCCAACTATTTC	**RThoxF1F**	AGTCAAAGCCGTACAAACAG
**BDhupLR1**	CCAAGCGGTATCTAATGC	**RThoxF2F**	CAGTCGTCAACTGTTAGTCGTTCG
**VNhoxWF1**	CTCAAAAAACTGTCTTAGTGTTGG	**RThoxY1F**	ACGATTTGGTTAGCGGGTTGT
**VNhoxWR1**	GCTAAACATTTCACATTGGGAA	**Sqhox5R1R**	ATCACAACCGCCTACCAAGA
**BDhupWF1**	CTTTGGCTGCGGGTCGTC	**Sqhox6F1R**	CAACACCCATCACGGTCT
**BDhupWR1**	GATAATGCTGCTGTTGAGGTGATG	**Sqhox8R1R**	TCTATGTCAACCCGCCCA
**LMS3'A^*b*^**	TCCCTTCCATGACCTCAAAC	**Sq8.2**	TGCGCCTGTGGTTGTCTACGATG
**LMH2B^*b*^**	GCCGCAAATTCCACAAACTCG	**Sq9RR1**	GGAACTCAACACAACACAG
**LMH4'A^*c*^**	GGCTATCTCCTTAACGAAGA	**Sq10F1F**	CGACAAGTTGAAGCAAGGAAG
**LMH4'BF^*b*^**	ATCTGCGTCAGTTAGCCCGA	**SqWF1**	CTGTATTTGTAAGAGTTGCC
**LMH5A^*b*^**	GTGCAACAGAAGCCAGTCGC	**SqWF2**	CGGCTTCATGGTTAAAGTC
		**SqWF3**	AGATCAGGAGGCGGATAAAC

^*a *^F or A (forward) and R or B (reverse) designations refer to primer orientations in relation to the frame of the gene.

^*b *^Leitão *et al*. [2].

^*c *^Schütz *et al*. [4].

^*d *^Ferreira *et al*. [1].

### Identification and sequencing of the *hox *genes

The regions upstream and downstream of the 2.7 kb containing *hoxYH*, previously sequenced [2], were obtained using the Universal GenomeWalker™ Kit (Clontech Laboratories, Inc., Palo Alto, CA). The digestions of genomic DNA with restriction endonucleases [*Dra*I (Amersham Biosciences, Buckinghamshire, UK), *Eco*RV, *Hin*cII, *Hpa*I (MBI Fermentas, Burlington, Canada) and *Xmn*I (New England Biolabs, Inc., Ipswich, MA)] were carried out overnight (16–18 h) at the temperatures recommended by the manufacturers. The DNA fragments were purified from the digestion mixture using phenol-chloroform, and ligated to the GenomeWalker™ Adaptor. Subsequently, the fragments were used in PCR amplifications with the gene-specific primers (GW-, listed in Table 2) together with the supplied Adaptor primers and following the PCR profiles recommended by the manufacturer (Clontech Laboratories, Inc., Palo Alto, CA). The PCR products were purified, cloned into pGEM^®^-T Easy vector (Promega, Madison, WI), and further used to transform *E. coli *DH5α competent cells following the instructions of the manufacturer. Colonies were screened for the presence of the insert by colony PCR and subsequently grown overnight, in liquid LB medium supplemented with 100 μg/ml of ampicillin, at 37°C with shaking. Plasmid DNA was isolated from *E. coli *cultures using the GenElute™ Plasmid Miniprep Kit (Sigma-Aldrich, Saint Louis, MO), and sequenced at STAB Vida (Lisbon).

To identify and sequence *L. majuscula*'s *hoxW*, the primer pair LahoxWF1-LahoxWR1 (based on *L. aestuarii*'s sequence, GenBank Accession number: L8106_07431) was used. The amplified PCR fragment was sequenced at STAB Vida (Lisbon). Further sequencing was achieved by the Genome Walking technique described above, using specific primers (GWhoxW-, listed in Table 2).

Published sequences were retrieved from GenBank and computer-assisted sequence comparisons were performed using Vector NTI Advance 10 (Invitrogen Corporation, Carlsbad, CA), and ClustalW [50]. Novel sequences associated with this study (*L. majuscula *CCAP 1446/4 *hoxEFUYH*, *hoxW*, and flanking ORFs) are available under the accession number [GenBank:AY536043].

### Cotranscription analysis (RT-PCR)

For the detection of *hox *transcripts, the RNA was extracted from cells grown under non-N_2_-fixing conditions (BG11_0 _+ ammonia) and collected at 6 h into the dark phase. For the detection of *hup *transcripts the RNA was extracted from cells grown under N_2_-fixing conditions (BG11_0_) and collected in the transition between the light and the dark phase. Reverse transcription (RT) reactions were performed with 1 μg of total RNA, following the protocol of the ThermoScript™ RT-PCR System (Invitrogen Corporation, Carlsbad, CA), and using LmhoxHR, GWhoxW1R or LmhupW2R as *hoxH*-, *hoxW*-, or *hupW*-specific antisense primers, respectively. The three different cDNAs produced were used as templates in PCR amplifications for the detection of the cotranscription of *hoxEF*, *hoxF-hcp*, *hoxUY, hoxYH *(cDNA generated using LmhoxHR), ORF16-*hoxW *(cDNA generated using GWhoxW1R), and *hupSL *and *hupL-W *(cDNA generated using LmhupW2R). The cDNAs produced were used in PCR amplifications performed with the primer pairs RThoxE1F-GWhox8R, RThoxF1F-LmHCPR, LMhoxUF1-GW5Lmhox2R, LmhoxYF-LmhoxHR, LmhoxWorfF1-LmhoxWR2, LMS3'A-LMH2B, and LMH5A-GW3LmhupWR1, for *hoxEF*, *hoxF-hcp*, *hoxUY*, *hoxYH*, ORF16-*hoxW*, *hupSL*, and *hupL-W *detection, respectively (Table 2). The PCR program profiles were as follows: 94°C for 2 min followed by 35 cycles of 45 s at 94°C, 45 s at 50°C (*hox*), 55°C (*hupSL*) or 64°C (*hupL*-*W*) and 1 to 2 min at 72°C, concluding with a 7 min extension at 72°C. Negative controls included the omission of reverse transcriptase in the RT reaction prior to the PCR, and a PCR to which no template was added. Genomic DNA was used as a positive control. Generated PCR products were analyzed on a 1% (w/v) agarose gel.

### Identification of transcription start points (tsp) by Rapid Amplification of cDNA Ends (5'-RACE)

The RNA used to establish the localization of the transcription start points was extracted from cells grown in the same conditions and collected at the same time points as for the cotranscription experiments (see above). 5'-RACE was carried out using the FirstChoice^® ^RLM-RACE Kit (Ambion, Inc., Austin, TX) following the instructions of the manufacturer. For the identification of the tsp upstream *hoxE*, *hoxW *and *hupW *the gene-specific antisense primers RChoxE1R, RChoxE2R, RChoxE3R, and RChoxE4R (*hoxE*), LmxisHR4, LmxisHR3, LmxisHR2, and LmxisHR1 (*xisH*), or LmhupW3R, LmhupW2R, LmhupW1R, and GW3LmhupWR1 (*hupW*) (Table 2) were used together with the kit adaptor-specific primers. PCR amplifications were carried out with the following profiles: 94°C for 3 min followed by 35 cycles of 30 s at 94°C, 30 s at 55°C (*hoxE *and *xisH*) or 58°C (*hupW*), and 1 min at 72°C, and concluding with 7 min extension at 72°C. The obtained PCR products were cloned into the pGEM^®^-T Easy vector (Promega, Madison, WI), and subsequently sequenced at STAB Vida (Lisbon).

### Transcription analysis by Real-time RT-PCR and RT-PCR

RNA was extracted from cells collected at six hours intervals during a complete 24 h light/dark cycle from cultures grown either under N_2_-fixing or non-N_2_-fixing conditions. For cDNA synthesis 1 μg of total RNA was transcribed with the iScript™ Select cDNA Synthesis Kit (Bio-Rad Laboratories, Inc., Hercules, CA), using the random primers supplied, and following the manufacturer's instructions. The PCR amplifications were performed using the primer pairs BDhoxHF1-BDhoxHR1, VNhoxWF1-VNhoxWR1, BDhupLF1-BDhupLR1, BDhupWF1- BDhupWR1, BD16SF1- BD16SR1 for *hoxH*, *hoxW*, *hupL*, *hupW*, and 16S rDNA detection, respectively (Table 2). For each analysis 16S rRNA gene was used for normalization. The PCRs (for Real-time analysis) were performed using 0.25 μM of each primer, 10 μl of iQ™ SYBR^® ^Green Supermix (Bio-Rad Laboratories, Inc., Hercules, CA) and 2 μl of template cDNA, while the PCRs for the RT-PCR assays were performed as described previously [48]. The PCR profile was: 3 min at 95°C followed by 50 cycles (Real-time RT-PCR) or 30 and 40 cycles (RT-PCR) of 30 s at 95°C, 30 s at 51°C and 30 s at 72°C. Standard dilutions of the cDNA were used to check the relative efficiency and quality of primers. Negative controls (no template cDNA) were included in all Real-time PCR and RT-PCR assays. A melting curve analysis was performed at the end of each Real-time PCR assay to exclude the formation of nonspecific products. Real-time PCRs were carried out in the ICycler iQ5 Real-Time PCR Detection System (Bio-Rad Laboratories, Inc., Hercules, CA). The data obtained were analyzed using the method described in Pfaffl [51].

## Authors' contributions

DF and FP performed the experimental work, PMF contributed to the discussion of this manuscript, MVM coordinated the Real-time PCR studies, PT conceived and coordinated this work and the manuscript. All authors read and approved the final manuscript.
